# Enhanced neuroinvasion by smaller, soluble prions

**DOI:** 10.1186/s40478-017-0430-z

**Published:** 2017-04-21

**Authors:** Cyrus Bett, Jessica Lawrence, Timothy D. Kurt, Christina Orru, Patricia Aguilar-Calvo, Anthony E. Kincaid, Witold K. Surewicz, Byron Caughey, Chengbiao Wu, Christina J. Sigurdson

**Affiliations:** 1 Departments of Pathology and Medicine, UC San Diego, La Jolla, CA USA; 2 Laboratory of Persistent Viral Diseases, Rocky Mountain Laboratories, National Institute of Allergy and Infectious Diseases (NIAID), National Institutes of Health (NIH), Hamilton, MT USA; 3 Departments of Biomedical Sciences, Medical Microbiology and Immunology, and Pharmacy Sciences, Creighton University, Omaha, NE USA; 4 Department of Physiology and Biophysics, Case Western Reserve University, Cleveland, OH USA; 5 Department of Neurosciences, UC San Diego, La Jolla, CA USA; 6 Department of Pathology, Immunology, and Microbiology, UC Davis, Davis, CA USA; 7 Current address: Division of Emerging and Transfusion-Transmitted Diseases, Office of Blood Research and Review, Food and Drug Administration, Silver Spring, MD USA

**Keywords:** Prion disease, Amyloid, Fibrils, Neurodegeneration, Prion strains, Axonal transport

## Abstract

Infectious prion aggregates can propagate from extraneural sites into the brain with remarkable efficiency, likely transported via peripheral nerves. Yet not all prions spread into the brain, and the physical properties of a prion that is capable of transit within neurons remain unclear. We hypothesized that small, diffusible aggregates spread into the CNS via peripheral nerves. Here we used a structurally diverse panel of prion strains to analyze how the prion conformation impacts transit into the brain. Two prion strains form fibrils visible ultrastructurally in the brain in situ, whereas three strains form diffuse, subfibrillar prion deposits and no visible fibrils. The subfibrillar strains had significantly higher levels of soluble prion aggregates than the fibrillar strains. Primary neurons internalized both the subfibrillar and fibril-forming prion strains by macropinocytosis, and both strain types were transported from the axon terminal to the cell body in vitro. However in mice, only the predominantly soluble, subfibrillar prions, and not the fibrillar prions, were efficiently transported from the tongue to the brain. Sonicating a fibrillar prion strain increased the solubility and enabled prions to spread into the brain in mice, as evident by a 40% increase in the attack rate, indicating that an increase in smaller particles enhances prion neuroinvasion. Our data suggest that the small, highly soluble prion particles have a higher capacity for transport via nerves. These findings help explain how prions that predominantly assemble into subfibrillar states can more effectively traverse into and out of the CNS, and suggest that promoting fibril assembly may slow the neuron-to-neuron spread of protein aggregates.

**Electronic supplementary material:**

The online version of this article (doi:10.1186/s40478-017-0430-z) contains supplementary material, which is available to authorized users.

## Introduction

Misfolded proteins incite cognitive and motor decline in Alzheimer’s, Parkinson’s, and prion disease. During a prion infection, prion aggregates, PrP^Sc^, template the misfolding of the cellular prion protein, PrP^C^, in an autocatalytic process that terminates in rapidly progressive and fatal neurodegeneration [1, 39]. Distinct PrP^Sc^ conformers drive PrP^C^ to misfold into a remarkable range of structural variants that correlate to profoundly different disease phenotypes [9]. Although most infectious prions spread from peripheral entry sites into the central nervous system (CNS), certain prion subtypes, such as variant Creutzfeldt-Jakob disease (vCJD), replicate and persist in lymphoid organs and fail to neuroinvade in mice [5, 14]. An estimated 1:2000 humans in the UK harbor vCJD prions in lymphoid tissues [20] and some individuals may remain lifelong subclinical carriers of extraneural infectious prions with no spread into the CNS. The physical properties of a prion aggregate that facilitate CNS entry and spread are unclear.

Peripheral nerves have been implicated as a major route for prion invasion of the CNS from extraneural entry sites, a process known as neuroinvasion. First, prions have been shown to spread from initial exposure sites into the brain by defined neuroanatomic pathways. For example, ingestion of prions induces early prion accumulation in the dorsal motor nucleus of the vagus and solitary tract nucleus in the brain as well as in the thoracic spinal cord, consistent with neuron-to-neuron spread via vagal and splanchnic nerve circuitry [4, 34]. Second, prions replicate in lymphoid tissues in early disease, prior to CNS invasion, and manipulation of the splenic innervation also indicates nerves as a possible conduit for prion trafficking into the CNS. For example, juxtaposing splenic nerves and prion-infected follicular dendritic cells (FDCs) [38], or increasing splenic innervation, accelerates prion spread to the brain in mouse models [21]. Finally, sympathectomy delays or prevents scrapie in mice [21], again suggesting an important role for peripheral nerves in prion neuroinvasion.

There are many details of protein aggregate spread in vivo that are not yet well understood, such as which PrP^Sc^ conformers transit in nerves. An advantage to investigating prion disease in mouse models is the highly reproducible incubation period and brain regions targeted by distinct prion conformational variants [18, 19], enabling studies linking PrP^Sc^ biophysical properties with disease phenotype. Although earlier studies demonstrated that certain prions show a limited capacity to spread via neurons into the CNS, the properties of prion aggregates that successfully traffic within nerves are unknown. We previously identified two mouse-adapted prion strains that failed to spread to the CNS following an intraperitoneal (IP) challenge of mice, and three prion strains that were highly neuroinvasive (NI) [7]. Both non-NI strains formed fibrillar, congophilic plaques in the brain after intracerebral (IC) inoculation, while the NI strains formed diffuse, noncongophilic aggregates that were less stable in chaotropes and lacked fibrils in the brain in situ, suggesting that a fibrillar structure correlated with poor neuroinvasion. Ultrastructural characterization of the fibrillar prions has been previously performed, and fibrillar prion strains were shown to form classical “kuru” type plaques composed of a central core of bundles [7, 25, 26, 28, 41]. Interestingly, GPI-anchorless prions form large fibrillar plaques and are also poorly neuroinvasive from peripheral exposure sites; in one study, the authors suggested that the GPI-anchor on the prion protein was important for prion transport via nerves [30]. Although some of these data may collectively argue that fibrillar prions show inefficient neuroinvasion, there is still no consensus on the physical requirements for prion entry into the CNS. Here we investigated the biophysical requirements for prion uptake and axonal transport in vitro using primary neurons, as well as prion neuroinvasion in vivo from a highly-innervated, extraneural exposure site, the tongue.

## Materials and methods

### Prion inoculation of mice with diverse strains

WT (VM/Dk) or *tg*a20 (Sv129/C57BL/6) mice (groups of 4-10 male and female mice, 2-3 months old) were inoculated intracerebrally into the left parietal cortex or intra-tongue with 10% or 1% prion-infected brain homogenate prepared from the brains of terminally ill mice. *Tg*a20 mice express the *Prnp* ^*a*^ sequence variant and develop prion disease after a short incubation period, for example, after IC inoculation, mCWD prions induce disease in 160 days in *tg*a20 mice, but more than 500 days in WT mice. VM/DK mice express the *Prnp* ^*b*^ sequence variant [35] and are highly susceptible to 87V fibrillar and 22L subfibrillar prion strains.

Mice expressing PrP under the NSE promoter, as well as *Prnp* ^*-/-*^ mice, were a kind gift from Dr. Adriano Aguzzi. NSE-PrP mice on a *Prnp* ^-/*-*^ background express more than 1.5-fold higher PrP^C^ than WT in cerebral hemispheres [22]. Mice were maintained under specific pathogen-free conditions on a 12:12 light/dark cycle (2 – 5 mice per cage) and were monitored three times weekly.

Strains 22L and ME7, as well as RML, are mouse-adapted prions originally derived from sheep scrapie that have different cellular targets in the brain and diverse plaque morphologies [11], and were kind gifts from Drs. Michael Oldstone and Adriano Aguzzi, respectively. Mouse-adapted CWD (mCWD) was derived from fifth passage of deer CWD in *tg*a20 mice [41].

TSE was diagnosed according to clinical criteria including ataxia, kyphosis, stiff tail, hind leg clasp, and hind leg paresis. Mice were sacrificed at early timepoints (50 and 75% of the incubation period) or at the onset of terminal disease. The brain was halved, and one hemi-brain was formalin-fixed for 2-3 days, then immersed in 96% formic acid for 1 h, washed in water, and post-fixed in formalin for 2-4 days. Brains were then cut into 2 mm transverse sections and paraffin-embedded for histological analysis. The remaining hemi-brain was cut and a 2-3 mm transverse section at the level of the hippocampus/thalamus was embedded in OCT and immediately frozen on dry ice. The remaining brain sections were frozen for biochemical analyses. No mice were excluded from the analysis.

### Histopathology and immunohistochemical stains

Four μm sections of brain were cut onto positively charged silanized glass slides and stained with hematoxylin and eosin or immunostained using antibodies for PrP (SAF84). For PrP staining, sections were deparaffinized and incubated for 5 min in 96% formic acid, then washed in water for 5 min, treated with 5 μg/ml of proteinase-K for 7 min, and washed in water for 5 min. Sections were then placed in citrate buffer (pH 6) and heated in a pressure cooker for 20 min, cooled for 5 min, and washed in distilled water. Sections were next incubated with anti-PrP SAF-84 (SPI bio; 1:400) for 45 min followed by anti-mouse biotin (Jackson Immunolabs; 1:250) for 30 min, followed by streptavidin-HRP (Jackson Immunolabs; 1:2000) for 30 min. Sections were then incubated with DAB substrate and an enhancer (Invitrogen), and counterstained with hematoxylin.

### Paraffin-embedded tissue (PET) blot

Five-μm thick sections were collected onto 0.45 μm nitrocellulose membranes (Biorad), dried at room temperature overnight, and heated at 55 °C for 30 min. Membranes were then incubated in xylene and serially rehydrated in 100%, 70% isopropanol, and distilled water with 0.1% Tween-20 for 10 min each. To improve tissue adherence, membranes were dried. After a brief rinse with TBST [10 mM Tris-HCl (pH 7.8), 100 mM NaCl, 0.05% Tween-20], membranes were incubated in 50 μg/ml of PK in 10 mM Tris-HCl (pH 7.8), 100 mM NaCl, 0.1% Brij-35 at 56 °C for 16 h, washed twice in TBST, incubated in 4M guanidine isothiocyanate in 10 mM Tris-HCl for 30 min, and washed in TBST. Membranes were blocked in casein (Sigma-Aldrich) and immunolabelled with anti-PrP monoclonal antibody SAF84 (Cayman Chemical) for two hours, biotinylated goat anti-mouse IgG (Jackson Immunolabs) for one hour, streptavidin-HRP (Jackson Immunolabs) for 30 min, and DAB substrate for 5 min. Color development was stopped by immersing briefly in distilled water and then membranes were dried overnight.

### Western blotting and sodium phosphotungstic acid precipitation

Brain tissue was homogenized in PBS using a Beadbeater™ tissue homogenizer. Homogenates in a Tris-based lysis buffer (10 mM Tris-HCl, 150 mM NaCl, 10 mM EDTA, 0.5% NP40, 0.5% DOC; pH 7.4) were digested with 50 μg/ml proteinase K at 37 °C for 30 min and the reaction stopped by boiling samples for 5 min in LDS loading buffer (Invitrogen). Samples were electrophoresed in 10% Bis-Tris gel (Invitrogen) and transferred to a nitrocellulose membrane by wet blotting. Membranes were incubated with monoclonal antibody POM19 (discontinuous epitope at C-terminal domain, amino acids 201–225 [37], a kind gift from Dr. Adriano Aguzzi) followed by incubation with an HRP-conjugated anti-mouse IgG secondary antibody (Jackson Immunolabs). The blots were developed using a chemiluminescent substrate (ECL detection kit, ThermoScientific) and visualized on a Fuji LAS 4000 imager. Quantification of PrP^Sc^ glycoforms was performed using Multigauge V3 software (Fujifilm).

PrP^Sc^ was concentrated from 87V and mCWD mouse brain samples by performing sodium phosphotungstic acid (NaPTA) precipitation prior to western-blotting [46]. Briefly, 100 μl aliquots of 10% brain homogenate in an equal volume of 4% sarkosyl in PBS were incubated for 30 min, then digested with an endonuclease [Benzonase™ (Sigma)] followed by treatment with 100 μg/ml proteinase K(50 μg/ml for WT brain) at 37 °C for 30 min. After addition of NaPTA, MgCl_2_, and protease inhibitors (Complete TM, Roche), extracts were incubated at 37 °C for 30 min, and centrifuged at 18,000 g for 30 min at 37 °C. Pellets were resuspended in 0.1% sarkosyl prior to electrophoresis and blotting.

### Prion uptake by primary neurons

Prions were partially purified by lysis in Tris buffered saline containing 2% sarcosyl, then were digested with an endonuclease for 30 min at 37 °C, and centrifuged at 18,000 g for 1 h. The pellets were washed and resuspended in PBS. Primary cortical neurons (200,000 cells) from E18 WT or *Prnp* ^*-*/*-*^ mouse embryos were cultured for a minimum of 6 days (in neurobasal media, 2% B27, and 1X GlutaMAX™) [51, 52]. In brief, the cerebral cortices were dissected, dissociated with 0.25% trypsin at 37 °C for 20 min, treated with DNase, and triturated. Debris was removed by passing the cells through a 40 μm cell strainer. Cells were then centrifuged for 5 min and resuspended in neurobasal media with 2% B27, 1X GlutaMAX™. Following several days in culture, neurons were then exposed to partially purified prions for timepoints from 0 - 48 h. At each timepoint, neurons were washed three times with cold PBS, treated with 0.25% trypsin for 3 min, centrifuged for 5 min at 2000 g, washed in cold PBS, and centrifuged again prior to cell lysis (10mM Tris-HCl, 150 mM NaCl, 1% sarcosyl). Total protein concentration was measured and equal protein amounts were assessed at each timepoint by western blot for analysis of prion uptake. Immunoblot signals were quantified using Multigauge V3 software (Fujifilm). To calculate the percent uptake, the signal at each timepoint was divided by the signal at the final timepoint, which was considered 100%. A minimum of three experimental replicates were performed.

### Exposure of neurons to compounds interfering with internalization

Cortical neurons from E18 mouse embryos were cultured for 7 days. Dynasore (80 μM), cytochalasin D (2 μM), amiloride (200 μM), 5-(N-ethyl-N-isopropyl)amiloride (EIPA) (50 μM), rottlerin (30 μM), chlorpromazine (5 μg/ml) in media were added to neurons for 30 min. Prions were then added to the neurons for 3 h, and then cells were washed three times with cold PBS and treated with 0.25% trypsin for 3 min to remove surface PrP^Sc^. Media was added and cells were collected and washed with PBS prior to lysis with lysis buffer (Tris-HCl, 150 mM NaCl, and 1% sarcosyl) and endonuclease treatment. Protein concentration was measured and proteins were normalized prior to proteinase K digestion and immunoblotting. Six experimental replicates were performed for all compounds except EIPA (3 replicates).

### Retrograde axonal transport using microfluidic chambers

Cortical neurons were cultured from wild type (C57BL/6) mouse E18 embryos. The cerebral cortices were dissected, dissociated with 0.25% trypsin at 37 °C for 20 min, treated with DNase, and triturated. Debris was removed by passing the cells through a 40 μm cell strainer. Cells were then centrifuged for 5 min and resuspended in neurobasal media with 10% FBS, 2% B27, 1X GlutaMAX™. Approximately 25,000 neurons were loaded into the cell body compartment of the polydimethylsiloxane microfluidic chamber for protein biochemistry assays [47]. After 5 min, the remaining compartments were filled with media. Cells were maintained in maintenance medium (neurobasal media with 2% B27 and 1X GlutaMAX™). The neurons were grown in the microfluidic chambers for 6 days or until neuronal projections extended into the axon compartment. Subfibrillar or fibrillar prions were added to the axon terminal compartment for 48 h. Prions were removed after 48 h by washing, and cell bodies and axons were collected 2 weeks later. The axons and somas were each washed three times with PBS. The soma chamber was washed by placing the chamber with the soma compartment in a vertical position and passing PBS through the somal well. The somas were collected first by similarly holding the chamber vertically and applying lysis buffer (10mM Tris-HCl, 150 mM NaCl, 1% sarcosyl, benzonase™, MgCl_2_) to the well and collecting the lysate. Axons were next collected by adding lysis buffer to the axon chamber. All chambers were assessed after use for leakage using trypan blue dye.

### RT-QuIC assay

RT-QuIC reaction mix was composed of 10 mM phosphate buffer (pH 7.4), 130 mM NaCl, 0.1 mg/ml recombinant mouse prion protein (residues 23-230 rPrP^Sen^), 10 μM thioflavin T (ThT), 1 mM ethylenediaminetetraacetic acid tetrasodium salt (EDTA), and 0.001% SDS. Aliquots of the reaction mix (98 μl) were loaded into each well of a black 96-well plate with a clear bottom (Nunc) and seeded with 2 μl of a 10^-1^ dilution of 22L, 87V or WT mouse brain-exposed neuronal lysates (somas or axons). The plate was sealed (plate sealer film, Nalgene Nunc International) and incubated at 42 °C in a BMG FLUOstar Omega plate reader with cycles of 1 min shaking (700 rpm double orbital) and 1 min rest. ThT fluorescence measurements (450 +/- 10 nm excitation and 480 +/- 10 nm emission; bottom read) were taken every 45 min. To compensate for minor differences in baselines between fluorescent plate readers and across multiple experiments, data sets were normalized to a percentage of the maximal fluorescence response (260,000 rfu) of the plate readers after subtraction of the baseline and plotted versus reaction time. Reactions were classified as RT-QuIC positive based on a threshold set by 1.5 standard deviations from the average of the wild-type control brains at 30 h (approximately 10% of ThT emission).

### Prion solubility assay of PrP^Sc^

Brain homogenates were solubilized in 1% sarcosyl in PBS and digested with 50 μg/ml of proteinase K (final) (WT) or 100 μg/ml (*tg*a20) for 30 min at 37 °C. Protease inhibitors were added (Complete TM™), and samples were layered over 15% Optiprep™ and centrifuged at 18,000 *g* for 30 min at 4 °C. Supernatants were removed and pellets were resuspended in PBS in a volume equivalent to the supernatant. Supernatant and pellet fractions were immunoblotted using anti-PrP antibody POM19 and PrP signals were captured and quantified using the Fuji LAS 4000 imager and Multigauge V3.0 software. Brain samples from 3-5 mice were measured per strain.

### PrP^Sc^ disaggregation assay

The protocol was adapted from Deleault et al., 2008 [15]. In brief, 10% brain homogenate was solubilized in 1% sarcosyl in PBS for 30 min at 37 °C. 1% Triton X-100 in PBS was added and samples were ultracentrifuged at 100,000 g for 1 h at 4 °C. The supernatant was discarded and pellets were resuspended in 1% Triton X-100 and incubated for 2 h at 37 °C. After 2 h, samples were subjected to sonication bursts of 10 s on and 30 s off at 80% power in a high intensity sonicator bath (Misonix™ horn sonicator) for a total sonication time of 3 min. Samples (700 μl) were then layered over 15% Optiprep™ (300 μl) and centrifuged at 10,000 g for 30 min at 4 °C. Pellets were resuspended in 0.2% sarcosyl in PBS. PrP^Sc^ in the supernatants was concentrated by sodium phosphotungstic acid (NaPTA) precipitation [46]. Samples were immunoblotted using the anti-PrP POM19 antibody, an HRP-conjugated anti-mouse IgG secondary antibody, and a chemiluminescent substrate, and signals were captured on the Fuji LAS 4000 Imager and measured using the Multi Gauge V3.0 software. Brain samples from 4-6 mice were measured per strain.

### Assessing the size of recombinant PrP fibrils

Recombinant PrP fibrils were prepared by expression and purification of mouse PrP (amino acids 23-144) followed by fibrillization as previously described [29]. Fibrils were sonicated the same way as the brain homogenates: 80% power, 10 s on, 30 s off, for a total of 3 min of sonication time. Negative stain electron microscopy was performed to compare the unsonicated and the sonicated fibril length. Fibrils were loaded onto a 100 mesh copper grid, washed with PBS, and stained with 2% uranyl acetate. Grids were imaged using transmission electron microscopy on a Tecnai G2 Spirit BioTWIN transmission electron microscope equipped with an Eagle 4k HS digital camera (FEI). For quantification, 8-12 isolated fibrils from 12 images (93 and 147 unsonicated and sonicated fibrils, respectively) were measured using ImageJ software (NIH). Sonicated and unsonicated fibril lengths were compared using a Student’s *t*-test.

### Prion fibril solubility assay

Fibrils were diluted to 0.4 mg/ml in PBS. Sonicated and unsonicated fibrils were layered over 15% Optiprep™ and centrifuged at 18,000 *g* for 30 min at 4 °C. Supernatants were removed and pellets were resuspended in PBS in a volume equivalent to the supernatant. Supernatant and pellet fractions were immunoblotted using anti-PrP antibody POM1 and PrP signals were captured and quantified using the Fuji LAS 4000 imager and Multigauge V3.0 software.

### Statistics

Data are presented as mean ± SEM unless otherwise indicated with group differences tested using standard parametric methods (Student’s *t*-test, 2-tailed). *P* values of less than 0.05 were considered statistically significant.

## Results

### Early entry and replication of subfibrillar prions in the brainstem after an intra-tongue prion exposure

**Fig. 1:**
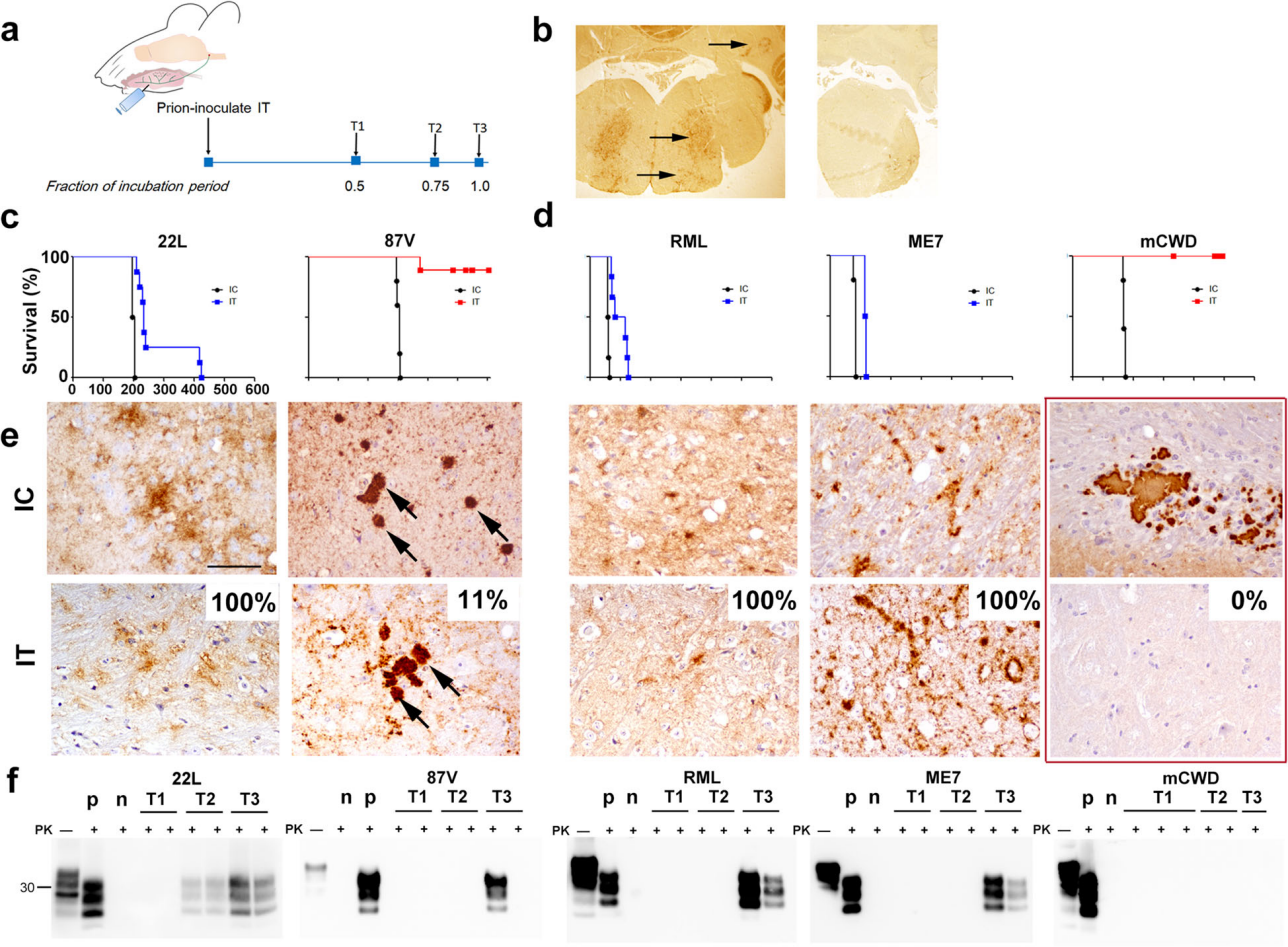
Fibrillar prion strains rarely neuroinvade following intra-tongue inoculation. **a** Schematic of time course experiment following IT exposure. T1, and T2: 0.50 and 0.75 of the expected incubation period; T3: terminal disease (1.0). **b** Paraffin-embedded tissue (PET) blot of brainstem (T2 timepoint) shows subfibrillar prions in the facial, reticular, and deep cerebellar nuclei (strain RML, left panel, arrows), whereas fibrillar prions were not detected at any early timepoint (strain 87V, right panel). **c** Survival curves of WT or **d** *tg*a20 mice (overexpress mouse PrP^C^) inoculated IT or IC with subfibrillar (blue - 22L, RML, ME7) or fibrillar (red - 87V, mCWD) prion strains. **e** Brain immunolabelled for PrP shows diffuse prion aggregates or large dense plaques in mice exposed to highly or poorly neuroinvasive prions, respectively. No plaques were detected in mice inoculated with mCWD IT (*red box* highlights brain sections from mice inoculated with mCWD by the IC versus IT route). Percentage of IT-inoculated mice that developed terminal prion disease is noted. Brain regions shown are as follows: cerebral cortex (IC: 22L); corpus callosum (IC: mCWD); thalamus (IC: 87V, RML, ME7; IT: 22L); hypothalamus (IT: ME7); brainstem (IT: 87V, RML, mCWD). **f** Immunoblots of brain from T1, T2, and T3 timepoints post IT inoculation. “p” and “n” refer to prion positive and negative brain samples (controls), and PK indicates proteinase K treatment. For IC inoculated mice, *n* = 4 (22L), 5 (87V, ME7, mCWD), or 6 (RML). For IT inoculated mice, *n* = 8 (22L), 9 (87V), 6 (RML), 4 (ME7), or 5 (mCWD). Scale bar = 100 μm

Distinct, sequence-matched PrP^Sc^ assemblies, or strains, are associated with remarkably varied clinical and pathologic disease phenotypes [10, 19]. We first examined the ability of diverse prions to spread from the tongue to the brain using a panel of strains comprising primarily fibrillar (87V, mCWD) or subfibrillar assemblies (amorphous oligomers or short fibrils) (22L, RML, ME7), which are defined by whether fibrils are visible ultrastructurally in the brain in situ [7, 26–28, 41]. The tongue is a natural route for prion entry through abrasions [3], is highly innervated, and provides a direct route for prion spread to the brain via cranial nerves independent of a lymphoid replication phase [2]. WT mice (VM/DK background) and *tg*a20 mice, which overexpress mouse PrP by 6-8 fold under the prion promoter [17], were used to investigate prion spread (*Methods* detail the mice and prion strains). After an intra-tongue (IT) injection, all three subfibrillar prions induced a rapid progression to terminal disease in 100% of mice, whereas neither fibrillar strain led to efficient prion spread to the CNS, with either 0% or 11% of mice developing prion disease (Fig. 1a-f). A time course revealed initial replication of subfibrillar prions in the brainstem (facial nucleus, reticular formation and deep cerebellar nuclei) (Fig. 1a-b) and lateral hypothalamus by 50% of the incubation period (59 days post-inoculation), consistent with transport from the tongue to the brain via cranial nerves and spread to the hypothalamus. Clinically negative mice had no histologically or biochemically detectable prions, even following sodium phosphotungstic acid precipitation [46] to increase the sensitivity of detection (Fig. 1f). The single mouse that developed prion disease following inoculation with 87V prions developed both plaques and diffuse PrP^Sc^ deposits, similar to mice inoculated by the IC route (Fig. 1e).

To exclude the possibility that PrP was replicating in FDCs within lymphoid tissue and then spreading to the brain, we inoculated mice that express PrP under the control of the neuron specific enolase (NSE) promoter with three prion strains by either the IT or IC route. We found that the subfibrillar prions, RML and ME7, spread from the tongue to the brain, indicating that prion replication in lymphoid tissue was not required for brain entry. The fibrillar prion, mCWD, once again did not spread from the tongue to the brain (Additional file 1: Figure S1).

### Neuronal uptake of the subfibrillar and fibrillar prion strains by macropinocytosis

**Fig. 2:**
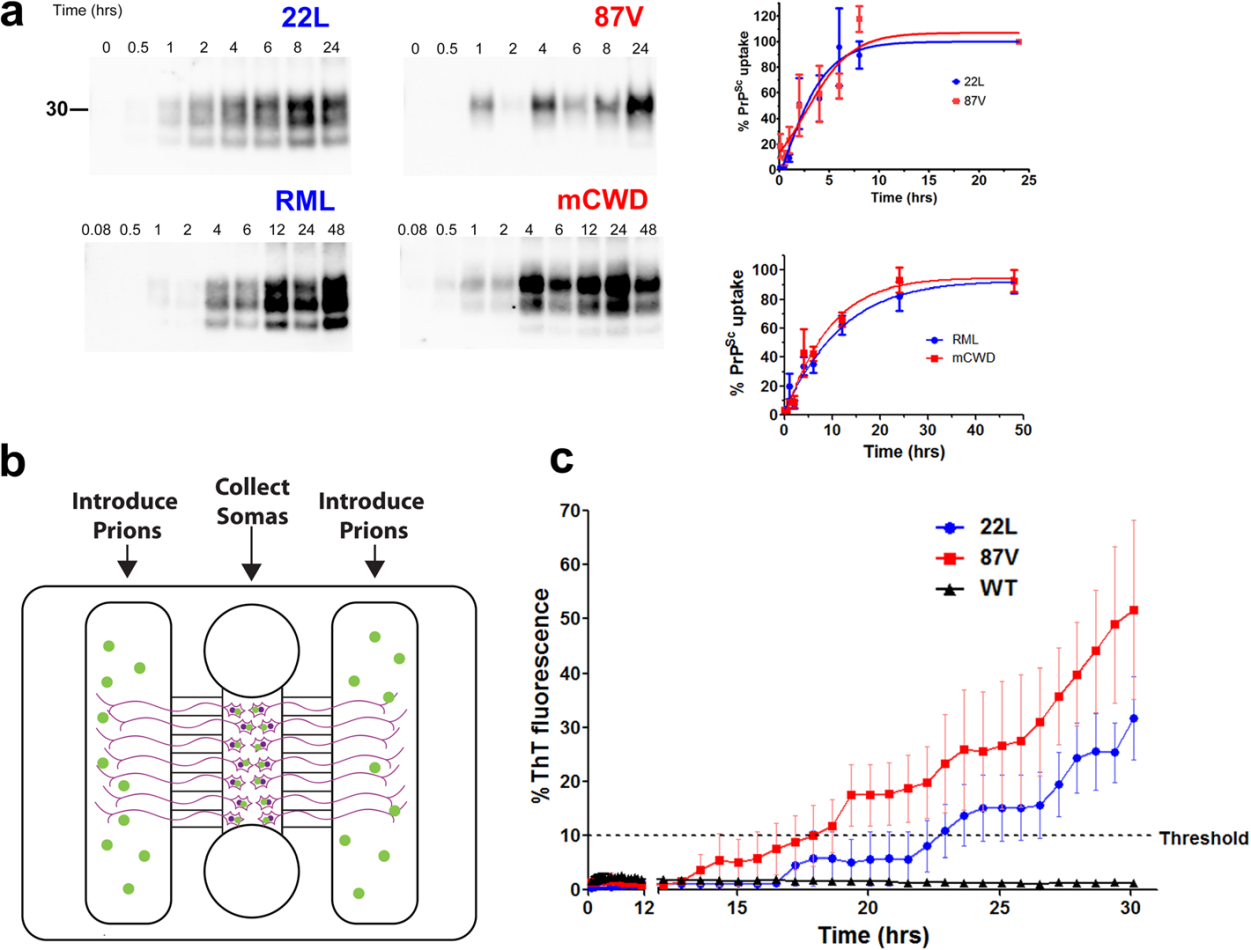
Subfibrillar and fibrillar prion strains are internalized and transported from the axon terminal to the soma. **a** Representative western blot shows subfibrillar (22L, RML) or fibrillar (87V, mCWD) prion internalization over time. Mean and SE from four (22L, 87V) or three (RML, mCWD) independent experiments. **b** Schematic of the microfluidic chamber in which the cells bodies reside in a large chamber and axons grow through fine grooves into side chambers where prions are introduced. **c** RT-QuIC analysis reveals PrP^Sc^ in the soma of neurons whose axons were exposed to subfibrillar (22L) or fibrillar (87V) prions, but not uninfected WT brain (WT). Shown are the mean and SE thioflavin T fluorescence signal for the positive cell body samples collected two weeks after prion exposure (4 of 8 positive samples per strain). The 87V prions were detected by RT-QuIC at slightly earlier times, which is not indicative of differences in the prion levels. Dashed line represents the threshold for positivity (see Methods)

To investigate the mechanism underlying the brain entry observed for the subfibrillar but not the fibrillar prion strains, we tested whether the neuronal uptake of prions correlated with the level of soluble, non-sedimenting prion particles, ie, the proteinase-K (PK)-resistant particles that remain suspended in 15% iodixanol following centrifugation. We first measured the non-sedimenting fraction of the five prion strains and found that the subfibrillar strains showed significantly more soluble, non-sedimenting particles than the fibrillar strains (*P* < 0.01) (Additional file 2: Figure S2). We next tested prion uptake by neurons. Using primary neurons, we found no difference in the internalization of subfibrillar or fibrillar prions (Fig. 2a, Additional file 3: Figure S3), and uptake was independent of PrP expression, consistent with previous reports [32]. As the mechanism of uptake may vary between the subfibrillar or fibrillar prion strains, macropinocytotic- and clathrin-mediated endocytic pathways were inhibited chemically. Macropinocytosis inhibitors markedly decreased the internalization of both subfibrillar and fibrillar prions, indicating that endocytosis of prions occurs primarily via macropinocytosis (Additional file 4: Figure S4). Inhibitors of clathrin-mediated endocytosis had less of an effect on prion uptake in primary neurons (Additional file 4: Figure S4).

### Axonal transport of prions from the axon terminal to the cell body

Given that neurons endocytose both subfibrillar and fibrillar prions in vitro, yet only the subfibrillar prions efficiently spread to the brain in vivo, we reasoned that prions may differ in their capacity for axonal transport. Therefore we next used neurons to examine the transit of prion aggregates from the axon terminals to the cell body. Neurons grown in microfluidic compartmentalized chambers were exposed to subfibrillar or fibrillar prions, or a mock control, for 48 h (Fig. 2b), and two weeks later the cell bodies were analyzed for PrP^Sc^ using the highly sensitive and specific real-time quaking induced conversion (RT-QuIC) assay [48]. As a control for leakage, microfluidic chambers lacking cells were exposed to prions in the axonal compartment, and the cell body compartments were assessed for prion seeding activity. As a further control, all chambers were assessed for leakage between compartments using trypan blue dye and showed no dye in the cell body compartment. Surprisingly, both the subfibrillar and fibrillar prion strains were detected in the cell bodies in equal numbers of experimental replicates (Fig. 2c). No prions were detected in the mock (Fig. 2c) or in the prion-seeded, cell-free control samples (data not shown). Collectively, these results suggest that the fibril-forming prion strains can transit in nerves, despite rare entry into the CNS in vivo.

### Increasing the particle number enhances neuroinvasion of 87V prions

**Fig. 3:**
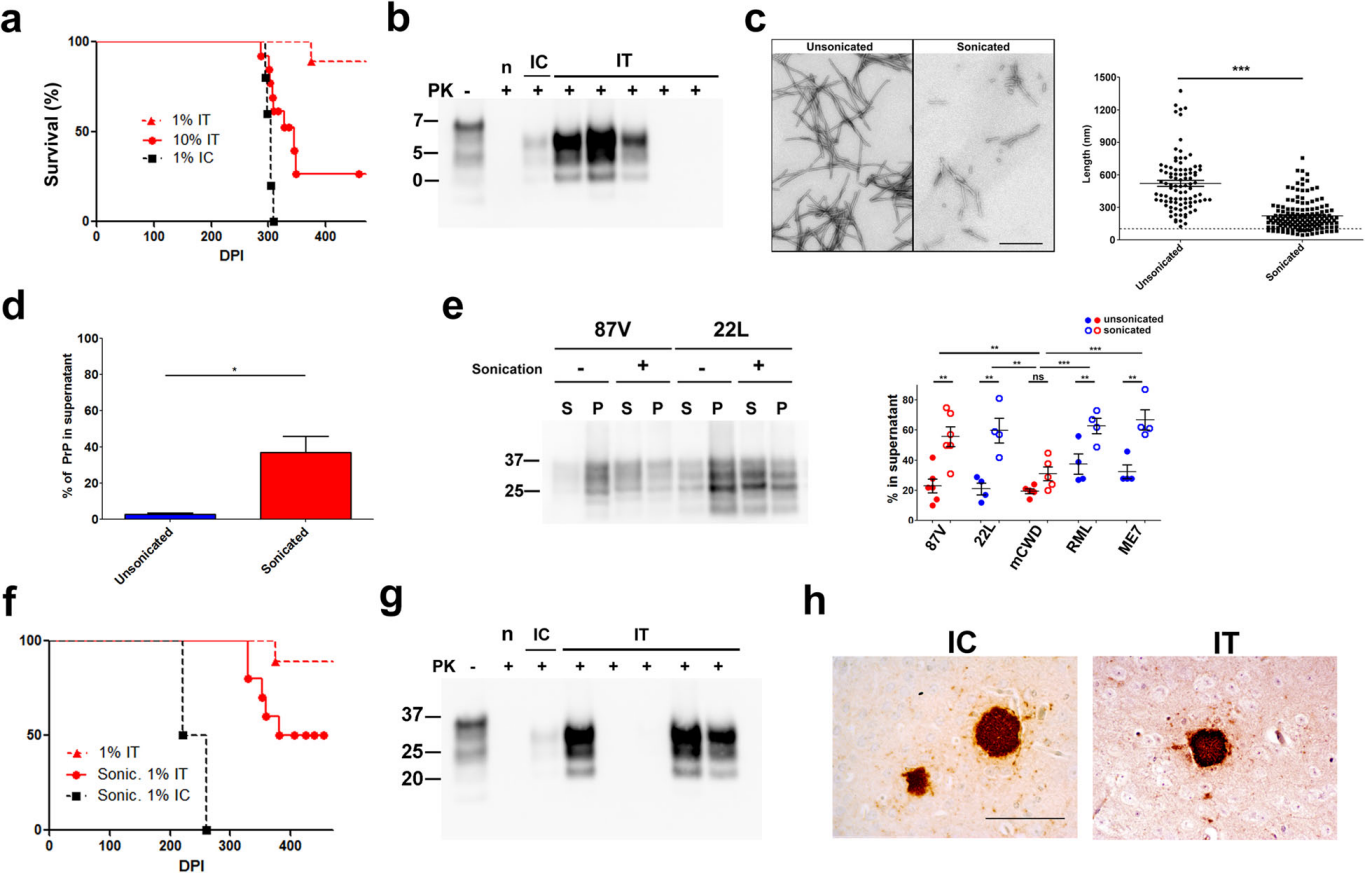
Increasing PrP^Sc^ concentration or sonicating prions increases neuroinvasion of fibrillar prion (strain 87V). **a** Survival curves of mice inoculated with a 10-fold higher concentration of fibrillar 87V prions (high dose: solid red line) (10% IT: *n* = 13; 1% IT: n = 9; 1% IC: *n* = 5) (1% IC and 1% IT mice are the same shown in Fig. 1c). **b** Representative western blot of brain shows proteinase-K (PK) resistant PrP^Sc^ in 3 of 5 mice exposed IT to high dose 87V prions. **c** Representative images of recombinant PrP fibril ultrastructure show that sonication results in short homogenous fibrils (mean length of unsonicated = 524 nm versus sonicated = 222 nm; *P* < 0.0001, Student’s *t* test). **d** Solubility assay on sonicated and unsonicated fibrils shows that sonication increases the solubility of the fibrils. **e** Disaggregation assay. Western blots show the increase of PK-resistant 22L and 87V prions in the supernatant following sonication of prion-infected brain homogenate. Quantification shows the results of all strains. **f** Survival curve of mice inoculated with sonicated, low dose (1%) fibrillar 87V prions. *n* = 10 (1% sonic. IT), 9 (1% IT), 4 (1% sonic. IC). The 9 mice inoculated with 1% prions IT are also shown in panel a and Fig. 1c. **g** Representative western blot of brain from mice exposed IT to sonicated fibrillar 87V prions shows PK-resistant PrP^Sc^ in 3 of 5 mice. **h** PrP^Sc^ shows large dense plaques in the brains of mice (cerebral cortex) exposed IC or IT to sonicated 87V prions. “*n*”: mock-inoculated brain control. Scale bars = 500 nm (**c**) and 100 μm (**h**)

Our findings indicate that the fibrillar prion strains are endocytosed and transported from the axon terminals to the neuronal cell bodies in vitro, although rarely enter the CNS in vivo. The in vitro exposure to the fibrillar strains would flood the axon terminals with small and large fibril fragments, whereas in vivo, the smaller, diffusible particles would likely travel faster through the interstitial space to the axon terminals [40]. To test the hypothesis that the level of small, diffusible prion particles impacts neuroinvasion, we exposed mice to a higher dose of 87V fibrillar prions IT (10-fold increase). We observed a 53% increase in the attack rate (number of mice developing terminal disease) and the presence of prion plaques in the cerebral cortex (Fig. 3a-b), indicating that the poor neuroinvasion was not due to an absolute block in the capacity for neuroinvasion, but that more prion particles or a higher titer, could enhance neuroinvasion of 87V prions.

### Increasing prion aggregate solubility enhances neuroinvasion

We and others have found that sonication decreases prion fibril size [13, 43], shifting the fibril population from a mixture of short and long fibrils to more uniformly short fibrils (Fig. 3c). In addition to decreasing the fibril size, sonication also markedly increased the solubility of prion fibrils from 3% soluble to approximately 37% soluble (Fig. 3d). We next assessed how sonication alters the solubility of brain-derived prions using a modified disaggregation assay that does not involve PK digestion to deplete the PrP^C^ [15]. In brief, samples were subjected to ultracentrifugation and the pellet fraction was resuspended and divided into two tubes, one of which was sonicated. All samples were then overlayed onto 15% iodixanol, centrifuged at 10,000 g, and the PrP^Sc^ was measured in the supernatant and pellet fractions. We found that the soluble, non-sedimenting fraction of all strains except mCWD significantly increased post-sonication (Fig. 3e). In the case that the number of small particles governs neuroinvasion, sonicating the prions while maintaining the same absolute prion mass would be expected to increase spread to the CNS. Sonicating the 87V fibrillar prions increased prions in the soluble fraction by 2.5 fold and significantly increased the attack rate, as 50% of mice developed terminal prion infection and prion plaques in the cerebral cortex (previously 11%). The mean incubation period was slightly shorter than the single mouse inoculated with the unsonicated prions (350 versus 374 days) (Fig. 3f). Our data suggest that a critical threshold of small, soluble particles is required for prion propagation into the CNS. Interestingly, the mCWD prions, which form exclusively large dense plaques of long fibrils [41] and primarily insoluble PrP^Sc^ aggregates, did not neuroinvade following exposure of mice to either a higher prion concentration or to sonicated prions (Additional file 5: Fig. S5). Collectively, these results show that high levels of small, soluble PrP^Sc^ particles correlates with the ability of a prion to neuroinvade.

## Discussion

Prion spread from tongue to brain is reported to be highly efficient, occurring rapidly and without requiring an initial replication phase in lymphoid tissue [2, 3, 6]. Consistent with these reports, we also found highly efficient, rapid spread of prions from tongue to brain for three subfibrillar strains. However, two fibrillar strains either failed to spread, or rarely spread following an intra-tongue challenge. Similar findings have been reported for other fibrillar prions inoculated into the tongue or other peripheral sites [5, 14], including GPI-anchorless prion fibrils, which accumulate in the nerves and muscle of the tongue, but fail to spread into the brain following a tongue inoculation [30].

The poor spread of fibrillar prions into the brain was unlikely due to a lack of prion uptake by neurons. In primary neurons, fibrillar prion strains were readily internalized by macropinocytosis, similar to other protein aggregates, such as SOD1 and tau [24, 49, 50]. Consistent with our findings, Magalhães and colleagues showed prion fibrils were internalized by neurons, and uptake was independent of PrP expression [32]. In addition to being internalized by neurons, the fibrillar 87V prion strain could transit from the axon terminal to the cell body in vitro, indicating that concentrated 87V prions applied directly to axon terminals could be transported within a neuron. This finding was consistent with the single mouse developing prion disease after intra-tongue inoculation, and indicates that the 87V prion strain can, in rare cases, transit into the CNS.

The failure of prion transport to the CNS in most mice exposed to 87V prions may be due to limited exposure of axon terminals to fewer small, soluble particles. When the PrP^Sc^ mass was held constant but the particle sedimentation, and presumably size, of 87V prions was reduced by sonication, prion neuroinvasion profoundly increased. Since sonication does not alter prion strain properties [15], the initial poor neuroinvasion of the fibrillar prions was not likely due to features of the quaternary structure, surface chemistry, or other biophysical properties of the 87V prions, but instead was likely due to the low number of small, non-sedimenting particles. Simply increasing the number of smaller, non-sedimenting prion particles, while maintaining the same PrP^Sc^ mass, enhanced neuroinvasion.

Was the increase in neuroinvasion simply due to a higher prion titer post-sonication? Smaller subfibrillar prion particles were shown to have more infectivity than larger fibrils when compared by mass of PrP [42]. Additionally, sonication likely increased the titer due to increasing the particle number and “free ends” available for recruiting PrP^C^ monomers, and we observed an 18% decrease in incubation period in IC-inoculated mice, consistent with a higher titer. However, if a high titer is the primary determinant for prion neuroinvasion, then GPI-anchorless RML fibrillar prions, which develop to very high titers in blood and heart of transgenic mice [45], should be highly neuroinvasive, even more so than their RML counterpart. Yet this is not the case, as GPI-prions are non-neuroinvasive by the intra-tongue route [30], although a requirement of a GPI-anchor for prion neuroinvasion cannot be excluded. High prion titers do not seem to strictly correlate with neuroinvasion.

Although titer does not correlate well with neuroinvasion, it is worth considering that a higher local prion titer in the tongue may increase prion replication locally and enable prions to neuroinvade. We and others have shown that prion neuroinvasion from the tongue does not require initial replication in local or distal lymphoid tissue. Additionally, mice exposed to the sonicated 87V prions showed an incubation period very similar to the mouse that developed infection from the non-sonicated prions (0.05% difference), suggesting that there was no major change in the incubation period due to any prolonged initial local replication phase. We would argue that the enhanced neuroinvasion observed with the sonicated 87V prions was not due to the increased number of “free ends” per se that enable a heightened peripheral replication phase, but was instead due to a higher number of smaller, soluble and diffusible particles that traverse the interstitial space for nerve entry and axonal transport.

Pathogenic prions form a spectrum of small subfibrillar to highly fibrillar aggregates. The findings reported here may be most relevant to the highly fibrillar, plaque-forming prions with few low density prion particles, similar to the non-neuroinvasive mCWD strain. Bovine amyloidotic spongiform encephalopathy (BASE) is thought to originate as a sporadic prion disease of cattle and causes dense, congophilic, fibrillar plaques in the brain [12]. BASE prions are highly infectious to cattle after an IC exposure [31]. Interestingly, an oral exposure of 16 cattle with 1-50 g of BASE prion-infected brain, containing 10^6.9^ LD_50_ / g, infected only one animal that had been exposed to 50 g of prion-infected brain (6% of exposed cattle) [36]. In this animal, there were fine and coarse PrP^Sc^ aggregates in the neuropil, but no fibrillar plaques in any brain section examined. Neither the second animal challenged with 50 g of brain, nor any other animal, developed clinical disease or any detectable PrP^Sc^. These findings suggest very rare entry of BASE prions into the CNS from an extraneural site, and only when the dose is exceedingly high. Similarly, variant CJD in 129M tg650 mice formed fibrillar plaques in the brain following IC inoculation, yet prions did not spread to the brain following intraperitoneal inoculation, despite early and persistent prion replication in the spleen [5]. Together with our findings, these studies suggest that fibril-rich, plaque-forming strains are inefficient at neuroinvasion. Since at least some plaque forming strains replicate in peripheral lymphoid tissues, the lack of neuroinvasion of such subtypes may lead to persistent subclinical carriers of infectious prions.

## Conclusion

Taken together, these findings support a model in which small, soluble prion particles shuttle between extraneural organs and the CNS via peripheral nerves. Slowly sedimenting prion particles were previously found to be highly infectious and a feature of strains that induce a rapidly lethal disease [44]. Future studies may indicate whether an abundance of small, more soluble particles distinguishes the highly infectious prions from amyloids such as amyloid-β and α-synuclein, which transit poorly from the eye or tongue into the CNS, respectively [8, 16]. These findings also suggest that therapeutic strategies designed to stabilize fibrils [23, 33] may hinder the neuronal transport of prions, and more generally other protein aggregates, thereby slowing the progression of neurodegenerative disease.

## Additional files

Additional file 1: Figure S1. (a) Survival curves of tg(NSE-PrP) mice inoculated IC or IT with RML, ME7, or mCWD prions. *N =* 4 mice per group for all groups except ME7 and mCWD IT where *n =* 5 mice. (b) Immunoblots from RML-, ME7-, and mCWD-inoculated Tg(NSE-PrP) mice. “n”: uninfected brain control. PK: proteinase K. (TIFF 5 kb)

Additional file 2: Figure S2. (a) Western blots show the solubility of two prion strains, 22L (subfibrillar) and 87V (fibrillar). S: supernatant and P: pellet. (b) Quantification of the pellet fraction for all five strains: 87V and 22L in WT mice (*n =* 5 mice each) and mCWD, ME7, and RML in tga20 mice [*n =* 3 (mCWD) or 4 mice (ME7, RML)]. *P < 0.05 and **P < 0.01 for 87V versus 22L prions (Student’s unpaired, 2-tailed *t*-test) and for *tg*a20 mice (one-way ANOVA followed by Tukey multiple comparison test). (TIFF 5 kb)

Additional file 3: Figure S3. Membrane bound prions are removed by trypsin. Uninfected N2a cells were cooled to 4 °C for 10 min and then exposed to partially purified 87V prions for 45 min at 4 °C. Cells were then washed three times with cold PBS, exposed to 0.25% trypsin for 3 min, centrifuged for 5 min at 2000 g, and washed three times in cold PBS prior to cell lysis, proteinase K digestion, and immunoblotting for prion protein. (TIFF 1 kb)

Additional file 4: Figure S4. Prion uptake in neurons occurs primarily by macropinocytosis. Primary neurons were exposed to prions after chemically inhibiting macropinocytotic- (amiloride, EIPA, cytochalasin D, rottlerin) and clathrin-mediated (dynasore) endocytic pathways. (a) Western blot shows very low PrP^Sc^ in cells in which the macropinocytotic pathways were inhibited. Note that dynasore, an inhibitor of clathrin-mediated uptake, had little effect on 22L or 87V prions. (b) Quantification of PrP^Sc^ uptake relative to the no drug control. Chlorpromazine was toxic to the cells and was not quantified. ***P < 0.0001 for 22L and *P < 0.05 for 87V, repeated measures one-way ANOVA. Results from the Tukey multiple comparison on the raw data are shown on the figure. Six experimental replicates were performed for all inhibitors except for EIPA, which had three replicates. (TIFF 4 kb)

Additional file 5: Figure S5. mCWD fibrillar prions did not neuroinvade from the IT route following exposure to 10-fold higher concentration of PrP^Sc^. (a) Survival curves show that no mice died with detectable mCWD prion plaques. (b) Western blots show no PK-resistant PrP^Sc^ in mice inoculated by the IT route. (c) PrP immunohistochemical stains of brain sections from mice exposed IT to 10% mCWD or 10% 87V. n: mock-inoculated brain control. For mCWD mouse groups: *n* = 10 (1% sonic.), 5 (10% IT), 5 (1% IT), 4 (1% IC). The mice inoculated with 1% mCWD prions IT and IC are the same as those shown in Fig. 1C. Scale bar = 100 μm. (TIFF 5 kb)
