# Supplementary figures and images for: Enhanced neuroinvasion by smaller, soluble prions
Source: Acta Neuropathol Commun. 2017 Apr 21;5:32. doi: 10.1186/s40478-017-0430-z (PMC5399838; doi:10.1186/s40478-017-0430-z)

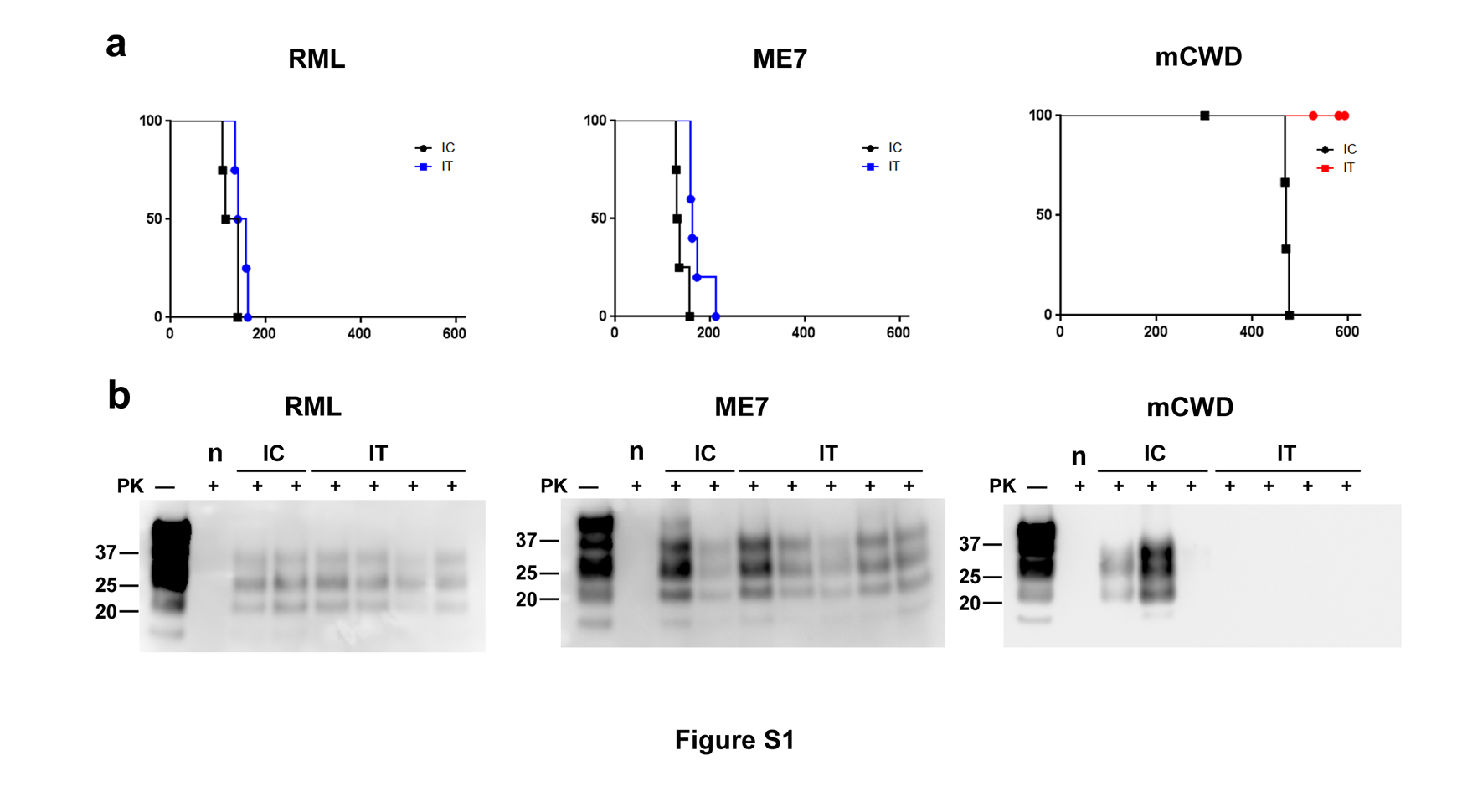

Supplement: Supplementary file 1 — (a) Survival curves of tg(NSE-PrP) mice inoculated IC or IT with RML, ME7, or mCWD prions. N = 4 mice per group for all groups except ME7 and mCWD IT where n = 5 mice. (b) Immunoblots from RML-, ME7-, and mCWD-inoculated Tg(NSE-PrP) mice. “n”: uninfected brain control. PK: proteinase K. (TIFF 5 kb) [file 40478_2017_430_MOESM1_ESM.tif]

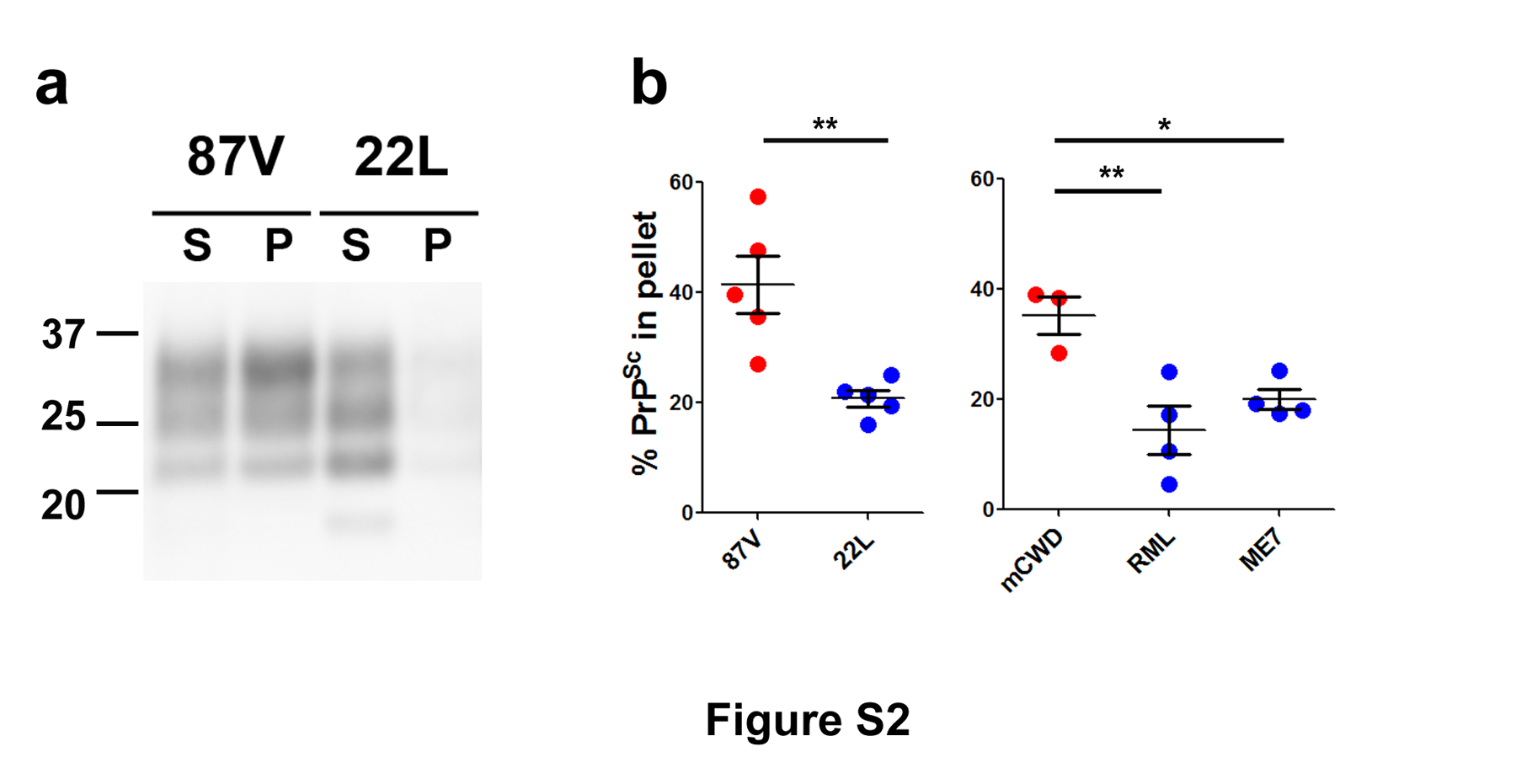

Supplement: Supplementary file 2 — (a) Western blots show the solubility of two prion strains, 22L (subfibrillar) and 87V (fibrillar). S: supernatant and P: pellet. (b) Quantification of the pellet fraction for all five strains: 87V and 22L in WT mice (n = 5 mice each) and mCWD, ME7, and RML in tga20 mice [n = 3 (mCWD) or 4 mice (ME7, RML)]. *P < 0.05 and **P < 0.01 for 87V versus 22L prions (Student’s unpaired, 2-tailed t-test) and for tga20 mice (one-way ANOVA followed by Tukey multiple comparison test). (TIFF 5 kb) [file 40478_2017_430_MOESM2_ESM.tif]

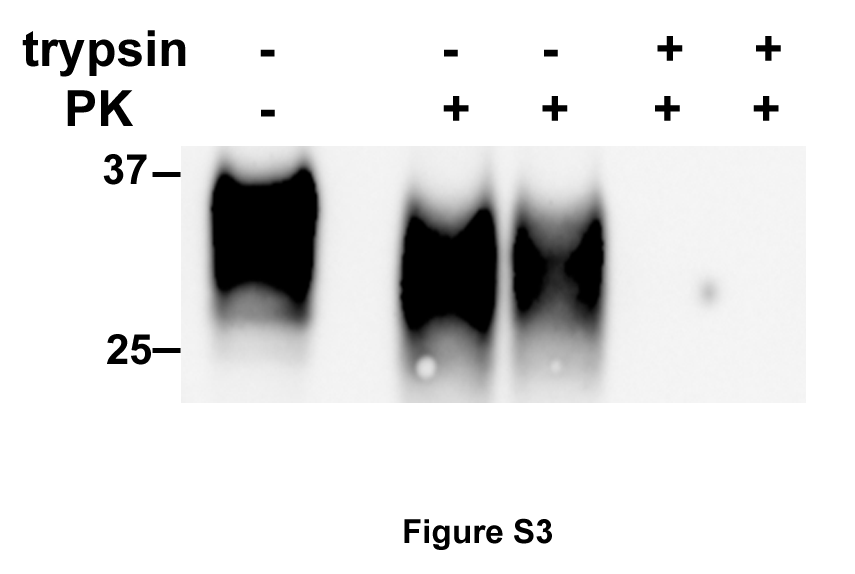

Supplement: Supplementary file 3 — Membrane bound prions are removed by trypsin. Uninfected N2a cells were cooled to 4 °C for 10 min and then exposed to partially purified 87V prions for 45 min at 4 °C. Cells were then washed three times with cold PBS, exposed to 0.25% trypsin for 3 min, centrifuged for 5 min at 2000 g, and washed three times in cold PBS prior to cell lysis, proteinase K digestion, and immunoblotting for prion protein. (TIFF 1 kb) [file 40478_2017_430_MOESM3_ESM.tif]

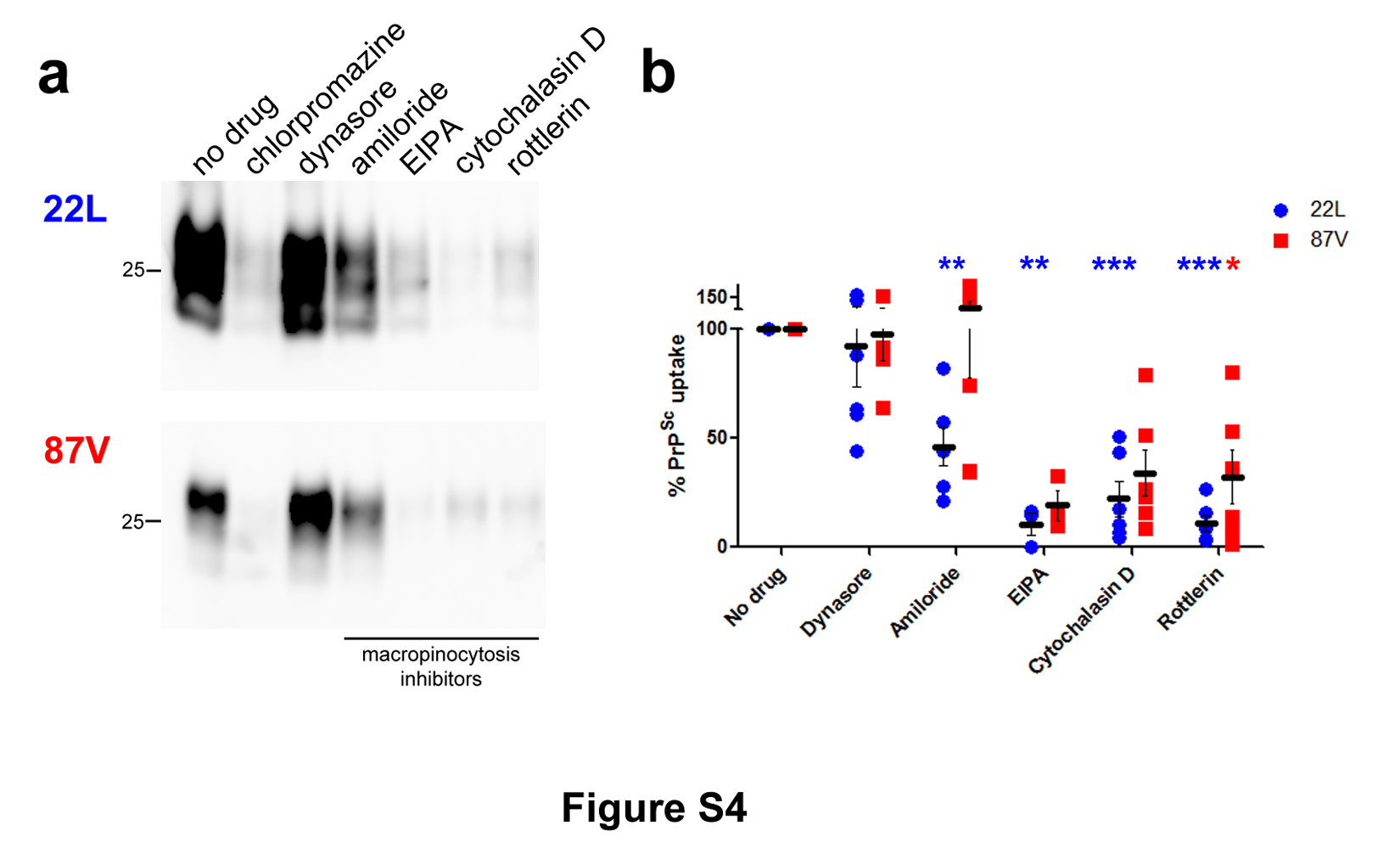

Supplement: Supplementary file 4 — Prion uptake in neurons occurs primarily by macropinocytosis. Primary neurons were exposed to prions after chemically inhibiting macropinocytotic- (amiloride, EIPA, cytochalasin D, rottlerin) and clathrin-mediated (dynasore) endocytic pathways. (a) Western blot shows very low PrPSc in cells in which the macropinocytotic pathways were inhibited. Note that dynasore, an inhibitor of clathrin-mediated uptake, had little effect on 22L or 87V prions. (b) Quantification of PrPSc uptake relative to the no drug control. Chlorpromazine was toxic to the cells and was not quantified. ***P < 0.0001 for 22L and *P < 0.05 for 87V, repeated measures one-way ANOVA. Results from the Tukey multiple comparison on the raw data are shown on the figure. Six experimental replicates were performed for all inhibitors except for EIPA, which had three replicates. (TIFF 4 kb) [file 40478_2017_430_MOESM4_ESM.tif]

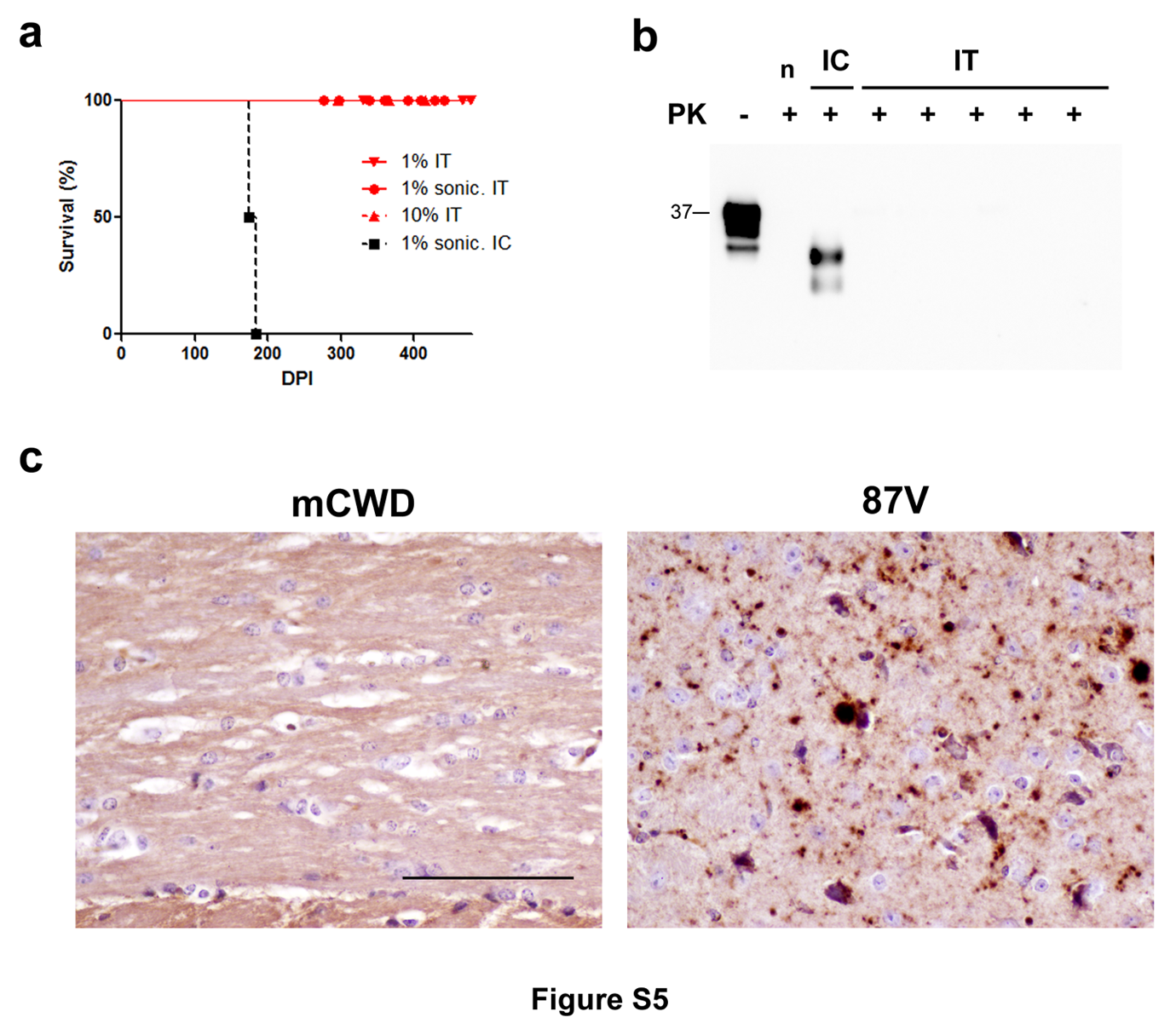

Supplement: Supplementary file 5 — mCWD fibrillar prions did not neuroinvade from the IT route following exposure to 10-fold higher concentration of PrPSc. (a) Survival curves show that no mice died with detectable mCWD prion plaques. (b) Western blots show no PK-resistant PrPSc in mice inoculated by the IT route. (c) PrP immunohistochemical stains of brain sections from mice exposed IT to 10% mCWD or 10% 87V. n: mock-inoculated brain control. For mCWD mouse groups: n = 10 (1% sonic.), 5 (10% IT), 5 (1% IT), 4 (1% IC). The mice inoculated with 1% mCWD prions IT and IC are the same as those shown in Fig. 1C. Scale bar = 100 μm. (TIFF 5 kb) [file 40478_2017_430_MOESM5_ESM.tif]
